# Circular RNA ZNF609 enhances proliferation and glycolysis during glioma progression by miR-378b/SLC2A1 axis

**DOI:** 10.18632/aging.203331

**Published:** 2021-09-14

**Authors:** Zhihuang Zhao, Gang Li, Yonggang Han, Yabin Li, Zhisheng Ji, Rui Guo, Xiaohong Yu

**Affiliations:** 1 Third Department of Neurosurgery, Cangzhou Central Hospital, Cangzhou, Hebei, China; 2 Bozhou Baozhang Hospital, Haozhou, Anhui Province, China; 3 Department of Urology Surgery, Linyi People’s Hospital, Linyi, Shandong Province, China

**Keywords:** glioma, proliferation, glycolysis, ZNF609, miR-378b, SLC2A1

## Abstract

Glioma is a prevalent brain malignancy with aggressive progression and with grave prognosis in adults. Circular RNAs have been reported to regulate glioma development and function as the diagnostic, prognostic, and therapeutic biomarkers. In this study, we were interested the function of circular RNA ZNF609 in modulating glioma. Remarkably, knockdown of ZNF609 by siRNA in glioma cells reduced cell viabilities and Edu-positive. The silencing of ZNF609 stimulated the apoptosis of glioma cells. Meanwhile, the ZNF609 depletion inhibited the invasion and migration of glioma cells. In glioma cells, the mRNA and protein expression of E-cadherin was enhanced, while Vimentin was reduced by the inhibition of ZNF609. The glucose uptake, lactate product, and ATP production in glioma cells were suppressed by ZNF609 knockdown. Mechanically, miR-378b was sponged by ZNF609 and targeted SLC2A1 in glioma cells. ZNF609 enhanced SLC2A1 expression by inhibiting miR-378b. The inhibition of miR-378b or the enhancement of SLC2A1 reversed ZNF609 depletion-regulated glioma cell proliferation *in vitro*. The depletion of ZNF609 suppressed glioma cell growth in the nude mice. Therefore, we concluded that ZNF609 contributed to cell survival and glycolysis of glioma by targeting miR-378b/SLC2A1 axis. ZNF609 and miR-378b may function as potential treatment targets in glioma.

## INTRODUCTION

Glioma is regarded as the most frequently occurring malignancy among brain tumors in adults, which is also highly aggressive and with grave prognosis [1]. Cancer development is a complicated process result from various aspects regarding proliferation, apoptosis, angiogenesis, and especially metabolism [2]. Over the past decade, the importance of metabolism has caused great attention in the cancer research area, due to the essential role of energy supplements in the maintenance of cell vitality [3]. Among the metabolism manners, enhanced glycolysis is proposed as an essential process for the initiation and development of cancer [4]. It is now well accepted that aerobic glycolysis is the major metabolic type in cancer cells, rather than the common oxidative phosphorylation in normal cells, even with the existence of oxygen, and this phenomenon is named the Warburg effect [5]. Noteworthy, glycolysis is recently suggested to be involved in metastasis of multiple cancers, including pancreatic cancer, lung cancer, and breast cancer and so on [6–9]. As a highly metastatic cancer type, glioma is also reported to be associated with glycolysis, involving the proliferation, inflammation, migration, and immune response [10].

Circular RNAs (CircRNAs) is a type of non-coding RNAs with a special covalent loop structure, which is found over four year ago and initially thought to be of low abundance [11]. Until recently, a great number of circRNAs were identified in multiple cell types across various species via high-throughput sequencing techniques, and were found to be profoundly involved in cell functions [11]. CircRNAs were regarded as potential therapeutic targets for cancer treatment, due to their role in regulating microRNA (miRNA) functions and the following gene expression [12, 13]. Emerging evidence also demonstrated that circRNAs were related to pathogenesis of multiples cancer, and were enriched in extracellular fluid, which suggested their potential role as diagnostic markers for cancer [12]. Among the cancer-related circRNAs, CircZNF609 was proposed as an activator of cell proliferation and metastasis, and affected biological process of cancer cell through regulating miRNAs [14–17]. For example, circZNF609 is upregulated in rhabdomyosarcoma and promotes the G1/S progression [14]. Wu and Zhu et al. proposed that circZNF609 promoted growth and migration of colorectal cancer and nasopharyngeal carcinoma through miR-150-5p [15, 16].

MicroRNA (miRNA) is a representative type of noncoding RNAs, characterized by a short sequence with around 20 nucleotides, which has been profoundly studied in cancer research [18]. The commonly recognized function of miRNAs is through interacting with the 3′UTR regions of targeted mRNAs and hinders gene expression, which subsequently leads to the alteration of multiple cellular functions, including growth, migration and angiogenesis [18]. MiR-378b functions as a suppressor of liver fibrosis through regulating Gli3 expression [19]. However, its role in glioma, especially the glycolysis, is not clear. SLC2A1, the gene encoding GLUT1, plays an important role in tumorigenesis and tumor progression of various cancer, which is closely related to glycolysis [20, 21]. For examples, inhibition of SLC2A1 could impede the growth of RB1-positive triple negative breast cancer [22]. SLC2A1 has been indicated to be targeted by a certain number of miRNAs to mediate glycolysis in cancer, such as miR-148b in gastric cancer and miR-328 in colon cancer [23, 24].

In this study, we were interested in the function of circular RNA circZNF609 in the modulation of glioma, especially glycolysis during glioma progression. Given that SLC2A1 is a critical factor in the regulation of glycolysis, we explored the effect of circZNF609 on SLC2A1 and the potential interaction of miR-378b with circZNF609 and SLC2A1 was screened by the bioinformatics analysis in the ENCORI database from several potential miRNAs. In our present study, we demonstrated a pro-metastatic role of circZNF609 in glioma, determined an interaction between miR-378b and SLC2A1, and established the role of this regulatory axis in circZNF609-mediated glycolysis during glioma progression. Our work may provide a new therapeutic target for glioma treatment.

## RESULTS

### The depletion of ZNF609 reduces glioma cell survival *in vitro*

**Figure 1:**
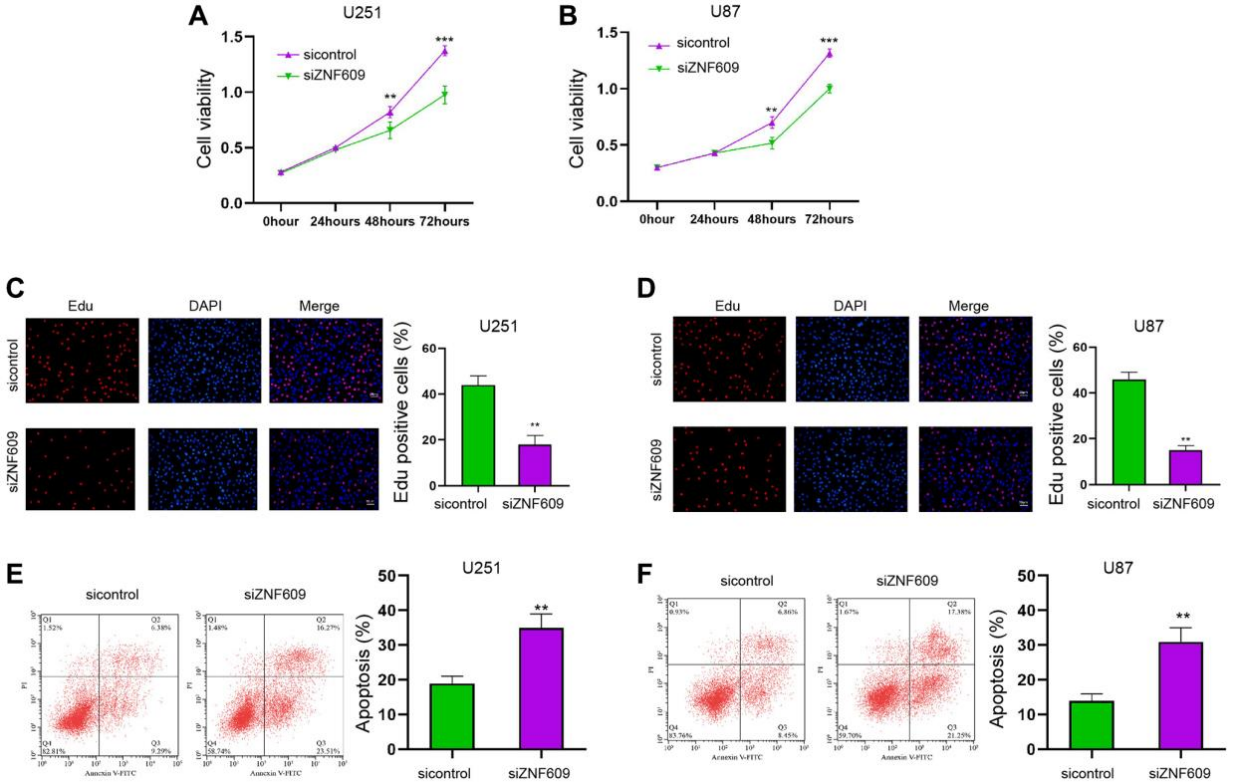
**The depletion of ZNF609 reduces glioma cell survival *in vitro*.** (**A**–**F**) The U251 and U87 cells were transfected with ZNF609 siRNA. (**A** and **B**) The cell viabilities of were analyzed by MTT assays. (**C** and **D**) The cell proliferation was detected by Edu assays. (**E** and **F**) The cell apoptosis was detected by flow cytometry. mean ± SD, ^**^P < 0.01.

Initially, ZNF609 was knockdown by siRNA in the U251 and U87 cells and the effectiveness of ZNF609 siRNAs was validated in the cells, in whichZNF609 siRNA-1 presented a higher efficiency and was selected in the subsequent analysis (Supplementary Figure 1A and 1B). Significantly, the knockdown of ZNF609 repressed U251 and U87 cell viabilities (Figure 1A and 1B). Similarly, the Edu-positive U251 and U87 cells were decreased by ZNF609 depletion (Figure 1C and 1D). Meanwhile, the silencing of ZNF609 stimulated apoptosis of U251 and U87 cells (Figure 1E and 1F).

### The depletion of ZNF609 represses glioma cell invasion *in vitro*

**Figure 2:**
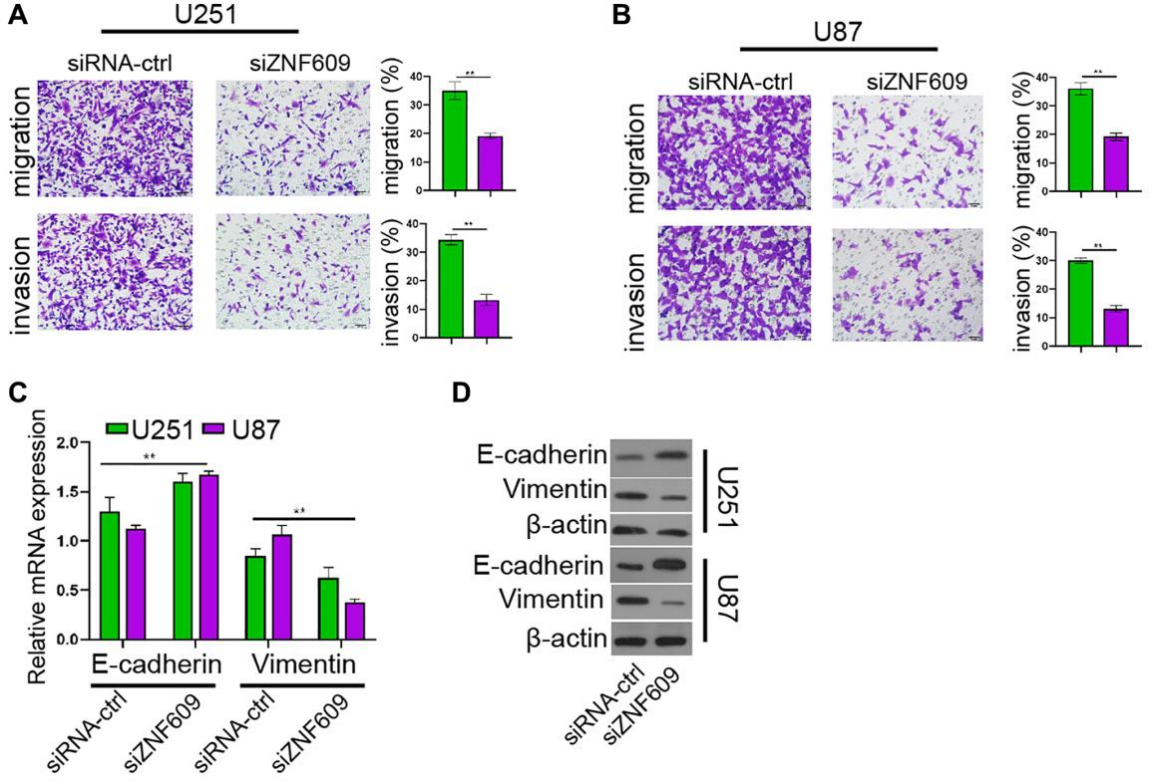
**The depletion of ZNF609 represses glioma cell invasion *in vitro*.** (**A**–**D**) The U251 and U87 cells were transfected with ZNF609 siRNA. (**A** and **B**) The cell invasion and migration were detected by transwell assays. (**C**) The mRNA of E-cadherin and Vimentin was analyzed by qPCR. (**D**) The protein levels of E-cadherin and Vimentin were determined by Western blot analysis. mean ± SD, ^**^P < 0.01.

Then, our data showed that the silencing of ZNF609 inhibited U251 and U87 cell invasion and migration (Figure 2A and 2B). Moreover, the depletion of ZNF609 enhanced E-cadherin mRNA expression and reduced Vimentin mRNA expression in U251 and U87 cells (Figure 2C). Consistently, the knockdown of ZNF609 increased E-cadherin protein expression while decreased Vimentin protein expression in U251 and U87 cells (Figure 2D).

### The depletion of ZNF609 inhibits glycolysis in glioma cells

**Figure 3:**
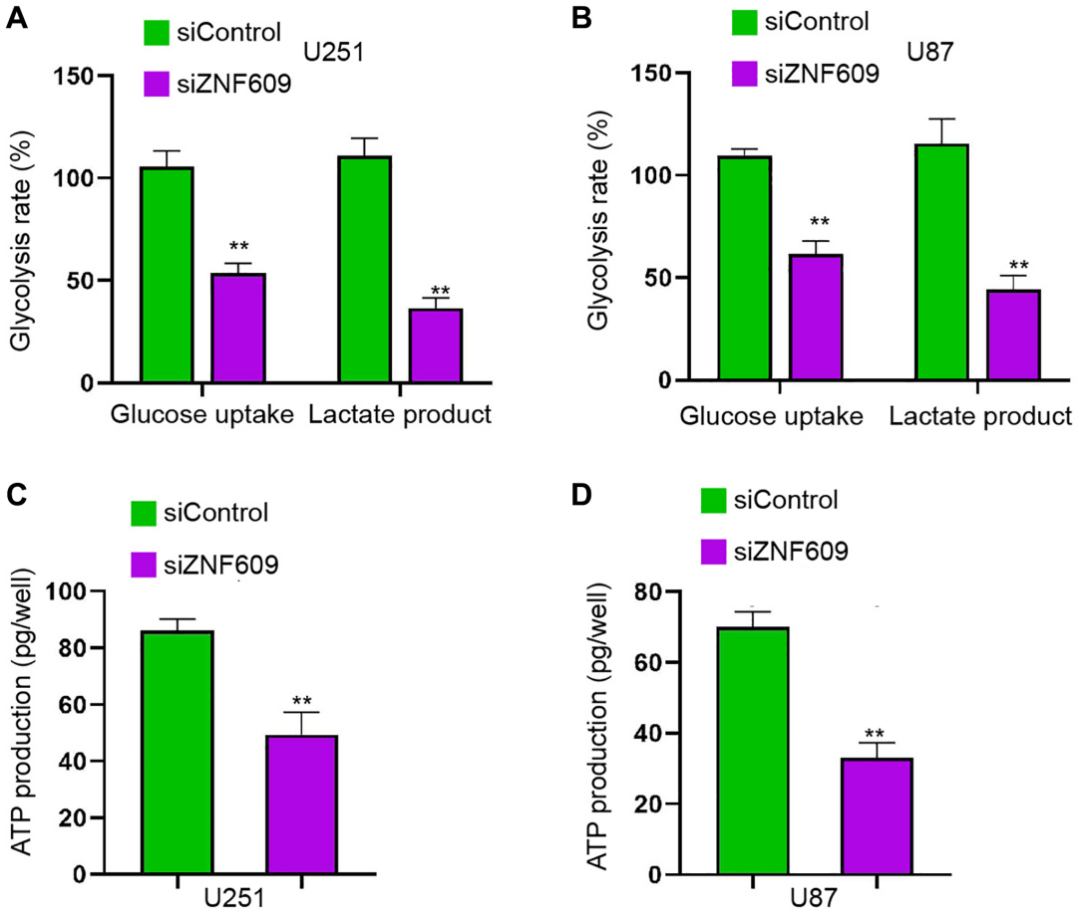
**The depletion of ZNF609 inhibits glycolysis in glioma cells.** (**A**–**D**) The U251 and U87 cells were transfected with ZNF609 siRNA. (**A** and **B**) The glucose uptake and lactate product were measured. (**C** and **D**) The ATP production was analyzed. mean ± SD, ^**^P < 0.01.

Then, we found that the silencing of ZNF609 repressed the glucose uptake and lactate product in U251 and U87 cells (Figure 3A and 3B). Consistently, the ATP production in U251 and U87 cells was reduced by the depletion of ZNF609 by siRNA *in vitro* (Figure 3C and 3D).

### ZNF609 is able to sponge miR-378b in glioma cells

**Figure 4:**
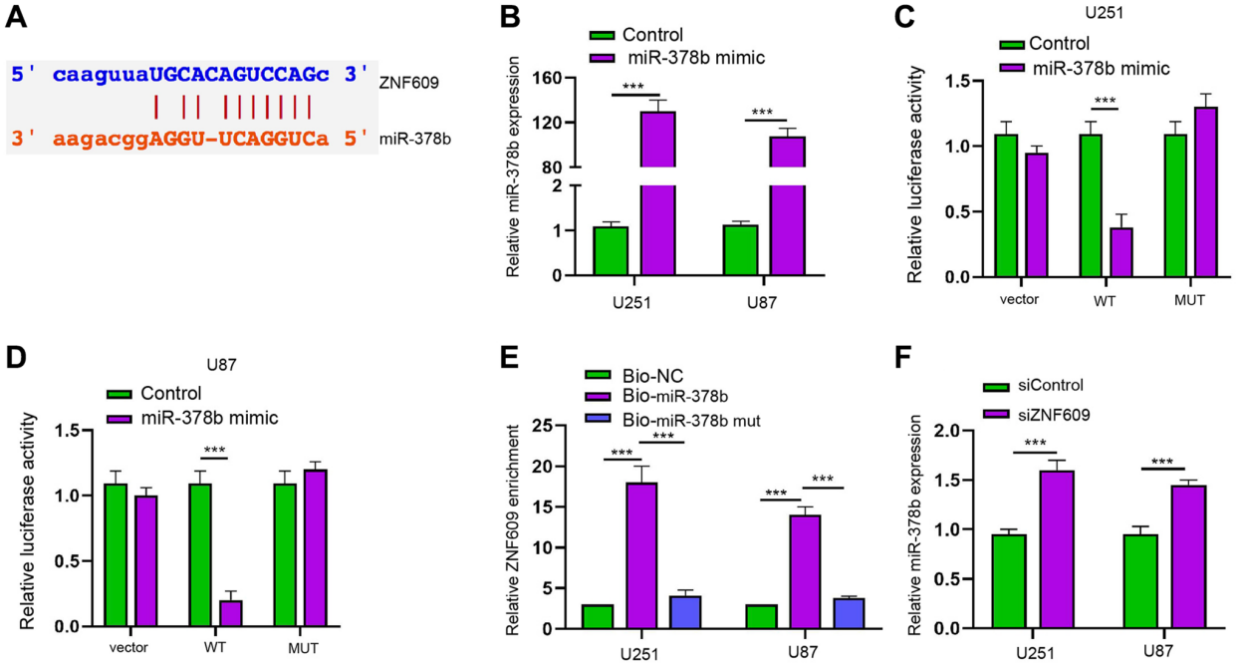
**ZNF609 is able to sponge miR-378b in glioma cells.** (**A**) The binding site prediction of ZNF609 and miR-378b in ENCORI database. (**B**–**D**) The U251 and U87 cells were treated with miR-378b mimic. (**B**) The expression of miR-378b was detected by qPCR. (**C** and **D**) The luciferase activity was detected by dual luciferase reporter assays. (**E**–**F**) The interaction of ZNF609 with miR-378b by RIP assays. [26] The expression of miR-378b was measured by qPCR in U251 and U87 cells treated with ZNF609 siRNA. mean ± SD, ^**^P < 0.01.

Then, the mechanical investigation identified the binding site of ZNF609 and miR-378b (Figure 4A). Meanwhile, the treatment of miR-378b mimic reduced luciferase activity of ZNF609 along with the enhanced miR-378b expression in U251 and U87 cells (Figure 4B–4D). RIP assays showed that miR-378b, but not miR-378b mutant, was able to directly interact with ZNF609 in U251 and U87 cells (Figure 4E). The silencing of ZNF609 by siRNA enhanced miR-378b expression in U251 and U87 cells (Figure 4F).

### MiR-378b is able to target SLC2A1 in glioma cells

**Figure 5:**
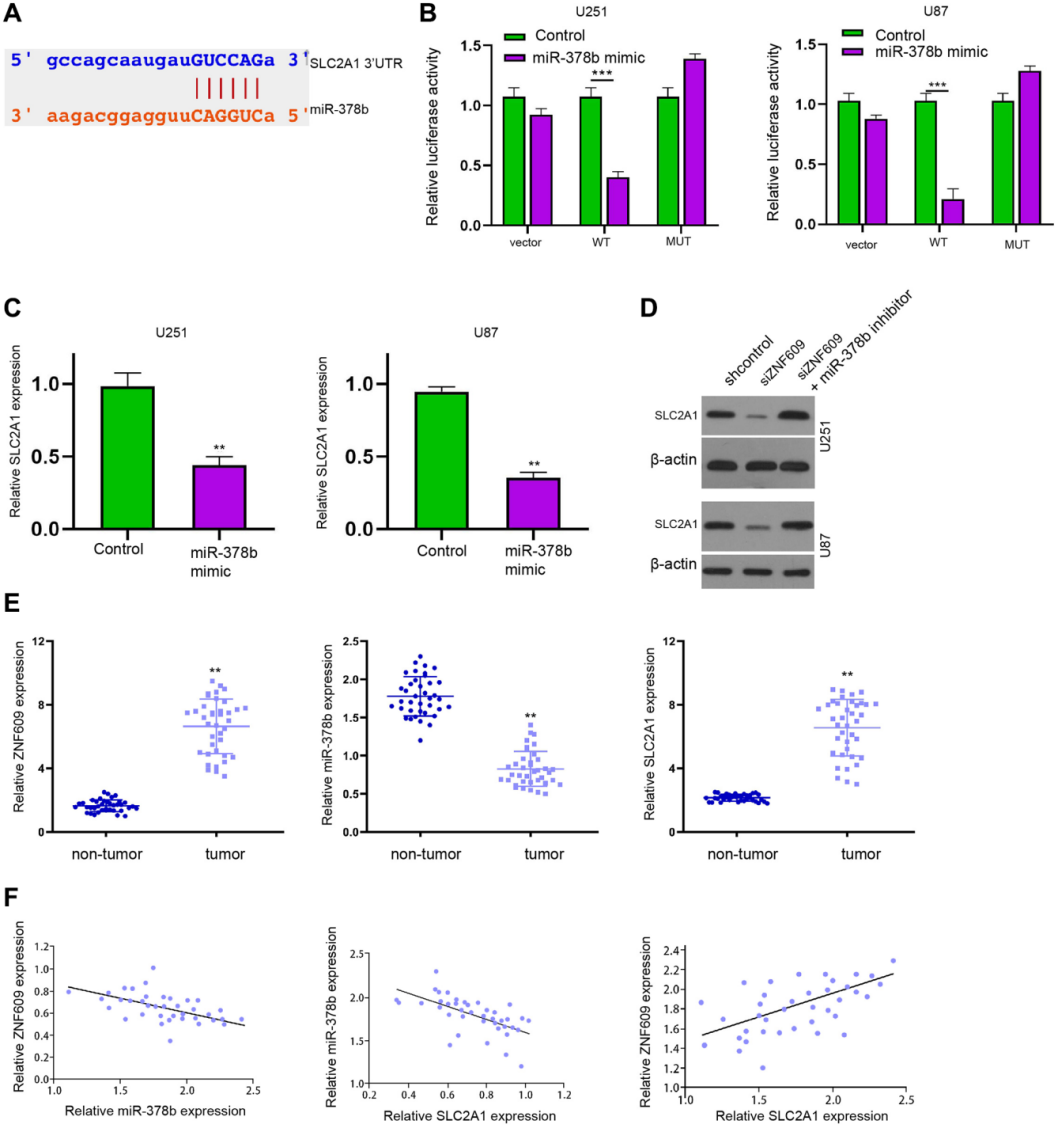
**MiR-378b is able to target SLC2A1 in glioma cells.** (**A**) The binding site prediction of SLC2A1 and miR-378b in ENCORI database. (**B** and **C**) The U251 and U87 cells were treated with miR-378b mimic. (**B**) The luciferase activity of SLC2A1 was measured by dual luciferase reporter assays. (**C**) The expression of SLC2A1 was analyzed by qPCR. (**D**) The expression of SLC2A1 was determined by Western blot analysis in U251 and U87 cells treated ZNF609 siRNA or co-treated with ZNF609 siRNA and miR-378b inhibitor. (**E** and **F**) The expression and correlation of ZNF609, miR-378b, and SLC2A1 were analyzed in the clinical glioma samples (*n* = 38). mean ± SD, ^**^P < 0.01.

We then predicted the binding site of miR-378b and SLC2A1 in a bioinformatic analysis (Figure 5A). Meanwhile, the treatment of miR-378b significantly suppressed luciferase activity of SLC2A1 mRNA 3′UTR in U251 and U87 cells (Figure 5B). Consistently, the treatment of miR-378b mimic reduced SLC2A1 expression in U251 and U87 cells (Figure 5C). The expression of SLC2A1 was repressed by ZNF609 silencing while miR-378b inhibitor rescued this repression in U251 and U87 cells (Figure 5D). The repression of miR-378b by miR-378b inhibitor was validated in U251 and U87 cells (Supplementary Figure 1C and 1D). Moreover, the ZNF609 and SLC2A1 expression were enhanced and miR-378b expression was reduced in the clinical glioma samples (Figure 5E). The expression of miR-378b was negatively correlated with ZNF609 and SLC2A1 and ZNF609 expression was positively associated with SLC2A1 in the clinical glioma samples (Figure 5F).

### ZNF609/miR-378b/SLC2A1 axis regulates progression of glioma cells

**Figure 6:**
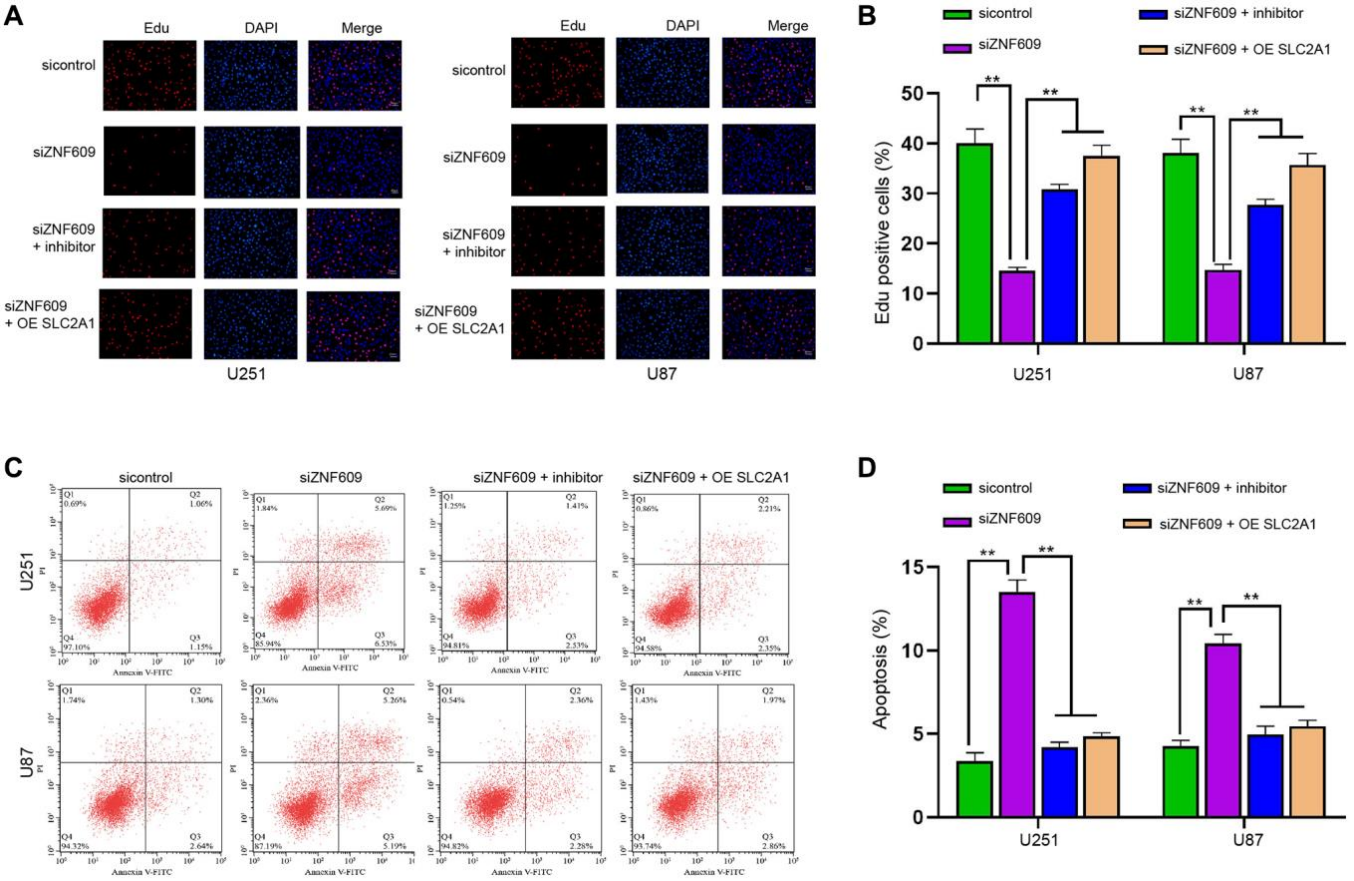
**ZNF609/miR-378b/SLC2A1 axis regulates progression of glioma cells.** (**A**–**D**) The U251 and U87 cells were treated with ZNF609 siRNA or co-treated with ZNF609 siRNA and miR-378b inhibitor or SLC2A1 overexpression vectors. (**A** and **B**) The cell proliferation was detected by Edu assays. (**C** and **D**) The cell apoptosis was detected by flow cytometry. mean ± SD, ^**^P < 0.01.

Next, our data showed that the depletion of ZNF609 inhibited the Edu-positive U251 and U87 cells, while the suppression of miR-378b or the enhancement of SLC2A1 could rescue the Edu-positive cells (Figure 6A and 6B). Then, the U251 and U87 cell apoptosis was induced by miR-378b knockdown, and the suppression of miR-378b or the enhancement of SLC2A1 blocked the effect (Figure 6C and 6D). The overexpression of SLC2A1 was validated in U251 and U87 cells (Supplementary Figure 1E and 1F).

### ZNF609 attenuates glioma cell growth *in vivo*

**Figure 7:**
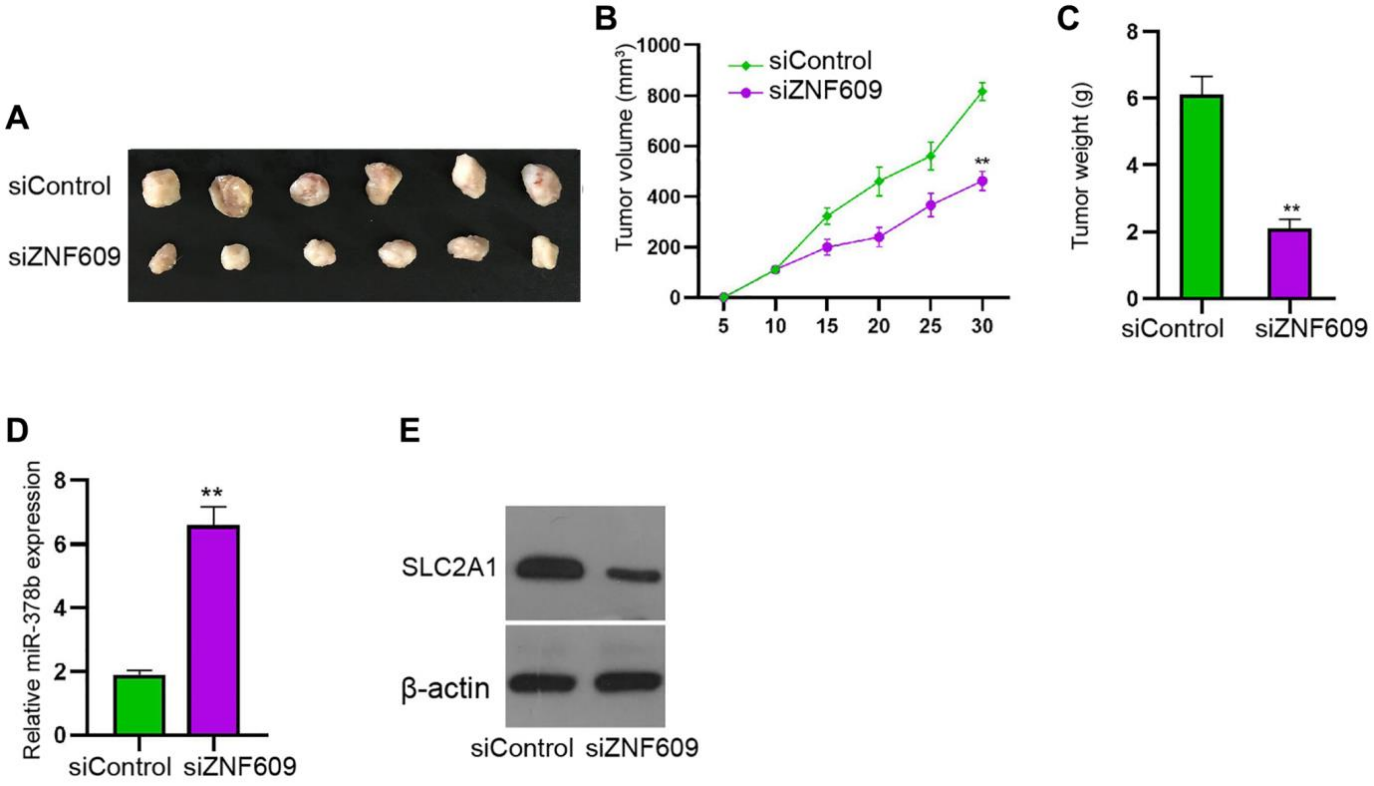
**ZNF609 attenuates glioma cell growth *in vivo*.** (**A**–**E**) The tumorigenicity analysis was conducted in nude mice injected with U251 cells treated with ZNF609 siRNAs. The tumor tissue images (**A**), tumor volume (**B**), and tumor weight (**C**) were demonstrated. The expression of miR-378b was measured by qPCR (**D**) and SLC2A1 expression was detected by Western blot analysis (**E**). mean ± SD, ^**^P < 0.01.

Next, tumorigenicity analysis in the nude mice injected with U251 cells showed that the silencing of ZNF609 by siRNA obviously attenuated the tumor growth of U251 cells in the nude mice (Figure 7A–7C). Moreover, the depletion of ZNF609 enhanced miR-378b and reduced SLC2A1 expression in the system (Figure 7D and 7E).

## DISCUSSION

Glioma is a prevailing brain cancers and circRNAs widely regulate glioma progression and have presented potential clinical values as diagnostic, prognostic, and therapeutic biomarkers. In this study, we were interested the function of circular RNA ZNF609 in modulating glioma.

It has been reported that circular RNA ZNF609 contributes progression of gastric cancer *via* repressing miRNA-145-5p [25]. ZNF609 promotes radioresistance by enhancing the glycolysis through miR-501-3p/HK2 signaling in prostate cancer [26]. ZNF609 contributes to proliferation of lung cancer cells by regulating miR-1224-3p/ETV1 axis [27]. Several circular RNAs have been reported to participate in glioma progression. Circular RNA PRKCI contributes to progression of glioma by repressing microRNA-545 [28]. Circular RNA TTBK2 modulates ferroptosis, invasion, and proliferation of glioma cells by miR-761/ITGB8 signaling [29]. Circular RNA CDR1as inhibits the p53/MDM2 signaling to reduce glioma progression [30]. These reports suggest that circRNAs play critical functions in the modulation of glioma development by regulating various cellular processes. In the present investigation, we found that the silencing of ZNF609 repressed cell survival, invasion, and glycolysis and stimulated cell apoptosis in glioma cells. The depletion of ZNF609 suppressed glioma cell growth in the nude mice. These data present the new function of ZNF609 in regulating glioma and provide new evidence of the role of circular RNAs in glioma development. In this study, we focus on the basic function of ZNF609 in the modulation of glioma, and the clinical value of ZNF609 is needed to explore in future studies.

MiR-378b has been reported to modulate cancer progression as a tumor suppressor. It has been reported that long Noncoding RNA CDKN2B-AS1 contributes to development of lung cancer by modulating miR-378b/NR2C2 axis [31]. Meanwhile, SLC2A1 plays an essential function in the regulation of glycolysis and progression of glioma. Long non-coding RNA LINC00174 enhances tumor progression and glycolysis in glioma *via* miR-152-3p/SLC2A1 axis [32]. N-acetylglucosaminyltransferase I enhances migration and proliferation of glioma cells by increasing SLC2A1 [33]. These reports indicate that SLC2A1 is a critical factor in controlling glycolysis and development of glioma. Our data showed that miR-378b was sponged by ZNF609 and targeted SLC2A1 in glioma cells. ZNF609 enhanced SLC2A1 expression by inhibiting miR-378b. The inhibition of miR-378b or the enhancement of SLC2A1 reversed ZNF609 depletion-regulated glioma cell proliferation *in vitro*. It implies that ZNF609 modulates glioma progression by miR-378b/SLC2A1 axis. MiR-378b and SLC2A1 may just one of the mechanisms of ZNF609-meidated glioma progression and other potential mechanisms are needed to investigate to comprehensively understand the function of ZNF609 in glioma. Meanwhile, the expression and correlation of ZNF609, miR-378b, and SLC2A1 were analyzed in this study. The correlation of ZNF609, miR-378b, and SLC2A1 and their function in other cancers should be explored by more investigations.

Therefore, we concluded that ZNF609 contributed to cell survival and glycolysis of glioma by targeting miR-378b/SLC2A1 axis. ZNF609 and miR-378b may function as potential treatment targets in glioma.

## MATERIALS AND METHODS

### Cell lines

Human glioma cell lines U251 and LN229 were purchased from the American Type Culture Collection (ATCC, USA), maintained in DMEM medium (Hyclone, USA) added with 10% FBS (Gibco, USA) and 1% penicillin/streptomycin (Sigma, USA), and were incubated in a 37°C atmosphere with 5% CO_2_.

### Clinical samples

The glioma samples (*n* = 38) were collected from glioma patients hospitalized, who received no chemotherapy and radiotherapy before surgery. All patients have signed the informed consent forms. All experiments were approved by the Clinical Ethics Committee of Cangzhou Central Hospital.

### Cell transfection

The SLC2A1 overexpressing vector (pCMV-SLC2A1) was constructed by cloning its cDNA sequence into the pCMV plasmid. The shRNA targeting circZNF609 (shZNF609), miR-378b mimics, miR-378b inhibitor or their corresponding negative controls (NC) were designed and obtained from Qiagene (USA). U251 and LN229 cells were cultured to form a 60% confluence, and lipofectamine 2000 was adopted to perform transfection for 24 hours. Then the cells were collected for further experiments.

### Cell proliferation and apoptosis assay

To obtain a cell vitality curve, U251 and LN229 cells were transfected with shZNF609 and the NC, followed by seeding in to 96-well plate (5000 cells per well). The Cell Counting Kit-8 (CCK-8, Beyotime, China) was adopted to determine the living cell numbers at 0 h, 24 h, 48 h, and 72 h, respectively. The values of absorbance at 450 nm were detected by a microplate spectrometer (PerkinElmer, Germany).

For detection of cells at S phase, 5-ethynyl-2′-deoxyuridine (EDU) assay was conducted under manufacturer’s instruction. In brief, U251 and LN229 cells after transfection were fixed and incubated with EdU reagent for 1 hour, followed by staining with Hoechst 33342 for 20 minutes. The positively stained cells were captured and counted using a fluorescence microscopy (Olympus, Japan).

To detect apoptotic cells, cells were digested by trypsin with no EDTA, and stained by a FITC-AnnexinV/PI apoptosis detection kit purchased from Beyotime, under the instruction of manufacturer.

### Transwell

The migration and invasion ability of U251 and LN229 cells were determined via using a transwell chamber (Corning, USA). To detect migration, U251 and LN229 cells (2 × 10^4^ cells/well) transfected with shZNF609 or the NC were seeded into the upper chambers with FBS-free medium, while the lower chambers were filled with complete DMEM medium. After 24 hours incubation, the membranes of upper chambers were fixed by 4% paraformaldehyde for 15 min, and stained by 0.5% crystal violet for 30 minutes. The migrated cells were photographed and counted. For cell invasion, the process was similar with that of migration experiment, only that the upper chambers were coated with Matrigel (BD Bioscience, USA).

### Western blotting

Ice-cold RIPA lysis buffer (Beyotime) was added to cells or tumor sections for extraction of total proteins. An equivalent 30 μg protein was separated via a SDS-PAGE and shifted to PVDF membranes. The blots were blocked in 5% non-fat milk for 1 hour, and incubated with primary antibodies against E-cadherin (1:500, Proteintech, China), Vimentin (1:500, Proteintech), SLC2A1 (1:1000, Proteintech), and GAPDH (1:1000, Proteintech) at 4°C overnight. Next day, the blots were soaked in HRP-conjugated secondary anti-mouse or anti-rabbit antibodies (1:1000, Abcam, USA). Then, an ECL kit (Thermo, USA) was used for the visualization of protein bands in a Gel imaging system (Bio-Rad, USA).

### Quantitative real-time PCR

Total RNAs were extracted from U251 and LN229 cells using the TRIzol reagent (Sigma) in accordance with the manufacturer’s instruction, reverse transcribed to cDNAs by using a TaqMan High-Capacity cDNA Reverse Transcription Kit (Thermo) for circZNF609, E-cadherin, Vimentin, and SLC2A1, or TaqMan MicroRNA Reverse Transcription kit (Thermo) for miR-378b. qPCR was performed by using a SYBR Green Reverse Transcription PCR kit (Thermo) in a 7500 Sequence Detection system. The relative expression of genes were calculated by the 2^−ΔΔCt^ method. The normalization of mRNA and miRNA used GAPDH and U6 as internal control. The primers were listed as follows: GAPDH, sense, 5′-TGTGGGCATCAATGGATTTGG-3′, antisense, 5′-ACACCATGTATTCCGGGTCAAT-3′; U6, sense, 5′-CGGGTGCTCGCTTCGCAGC-3′, antisense, 5′-CCAGTGCAGGGTCCGAGGT-3′; E-cadherin, sense, 5′-CGAGAGCTACACGTTCACGG-3′, antisense, 5′-GGGTGTCGAGGGAAAAATAGG-3′; Vimentin, sense, 5′-GACGCCATCAACACCGAGTT-3′, antisense, 5′-CTTTGTCGTTGGTTAGCTGGT-3′; miR-378b, sense, 5′-GGTCATTGAGTCTTCAAGG-3′, antisense. 5′-GGTCTTTCTGCCTCCA-3′; SLC2A1, sense, 5′-AAGGTGATCGAGGAGTTCTACA-3′, antisense, 5′-ATGCCCCCAACAGAAAAGATG-3′.

### Assessment of glycolysis biomarkers

The uptake of glucose, lactate secretion and ATP production were determined using the glucose assay kit (Betoyime), lactate assay kit (Sigma) and ATP detection kit (Beyotime) according to manufacturers’ protocols.

### Xenograft tumor model

All animal experiments in this work were authorized by Ethics Committee of Cangzhou Central Hospital. BALB/c nude mice aged 5-week were obtained from Laboratory Animal Center of Chinese Academy of Sciences (Shanghai, China), and maintained in a specific pathogen-free (SPF) environment. The mice were randomly divided into two groups (*n* = 5). A total number of 1 × 10^6^ U251 cells transfected with shZNF609 or NC were collected, washed, suspended in 100 μL saline, and then subcutaneously injected into the left fat pad of each mice. The tumor size (volume = 0.5 × width^2^ × length) and body weight were measured at the indicated time points.

### AGO2-RIP-PCR assay

The interaction between miR-378b and circZNF609 was analyzed by using RNA Immunoprecipitation kit (Millipore) following the manufacturer’s instructions. In brief, U251 and LN229 cells transfected with miR-378b mimics or the NC were collected and lysed. The lysates were added with beads conjugated with Ago2 or IgG antibodies. Then the beads were washed, and subjected to western blotting and qPRC for detection of circZNF609.

### RNA pulldown

U251 cells transfected with biotin-labeled miR-378b (RiboBio) were harvested 48 hours after transfection, and were lysed by specific lysate buffer (Thermo). The cell lysates were incubated with magnetic beads (Thermo) at 4°C for three hours. The beads were washed and subjected to qPRC to detect the enrichment of circZNF609.

### Dual luciferase reporter assay

The potential binding site of miR-378b with circZNF609 and SLC2A1 was screened using ENCORI database (http://starbase.sysu.edu.cn/index.php). The sequence of circZNF609 and 3′UTR region of SLC2A1 were cloned into the pmiR-Glo vector (Promega, USA) to obtained ZNF609-Wt and SLC2A1-Wt. Similarly, the specific mutagenesis at the binding site of miR-378b was adopted to obtain ZNF609-Mut and SLC2A1-Mut. U251 and LN229 cells were transfected with the WT or Mut along with miR-378b mimics or NC for 24 hours. The cells were lysed and the luciferase activity was measured by a dual luciferase detection kit (Promega).

### Statistics

Each data was obtained from at least three independent experiments, and was shown as mean ± SD. Statistical significances between two groups or multiple groups were determined by Student’s *t*-test or one-way ANOVA via a SPSS 19.0. *P* < 0.05 was considered statistically significant.

## Supplementary Materials

Supplementary Figure 1
