# Supplementary material for: Circular RNA ZNF609 enhances proliferation and glycolysis during glioma progression by miR-378b/SLC2A1 axis
Source: Aging (Albany NY). 2021 Sep 14;13(17):21122–33. doi: 10.18632/aging.203331 (PMC8457557; doi:10.18632/aging.203331)
Supplement: Supplementary Figure 1 [file aging-13-203331-s001.pdf]

## SUPPLEMENTARY FIGURE

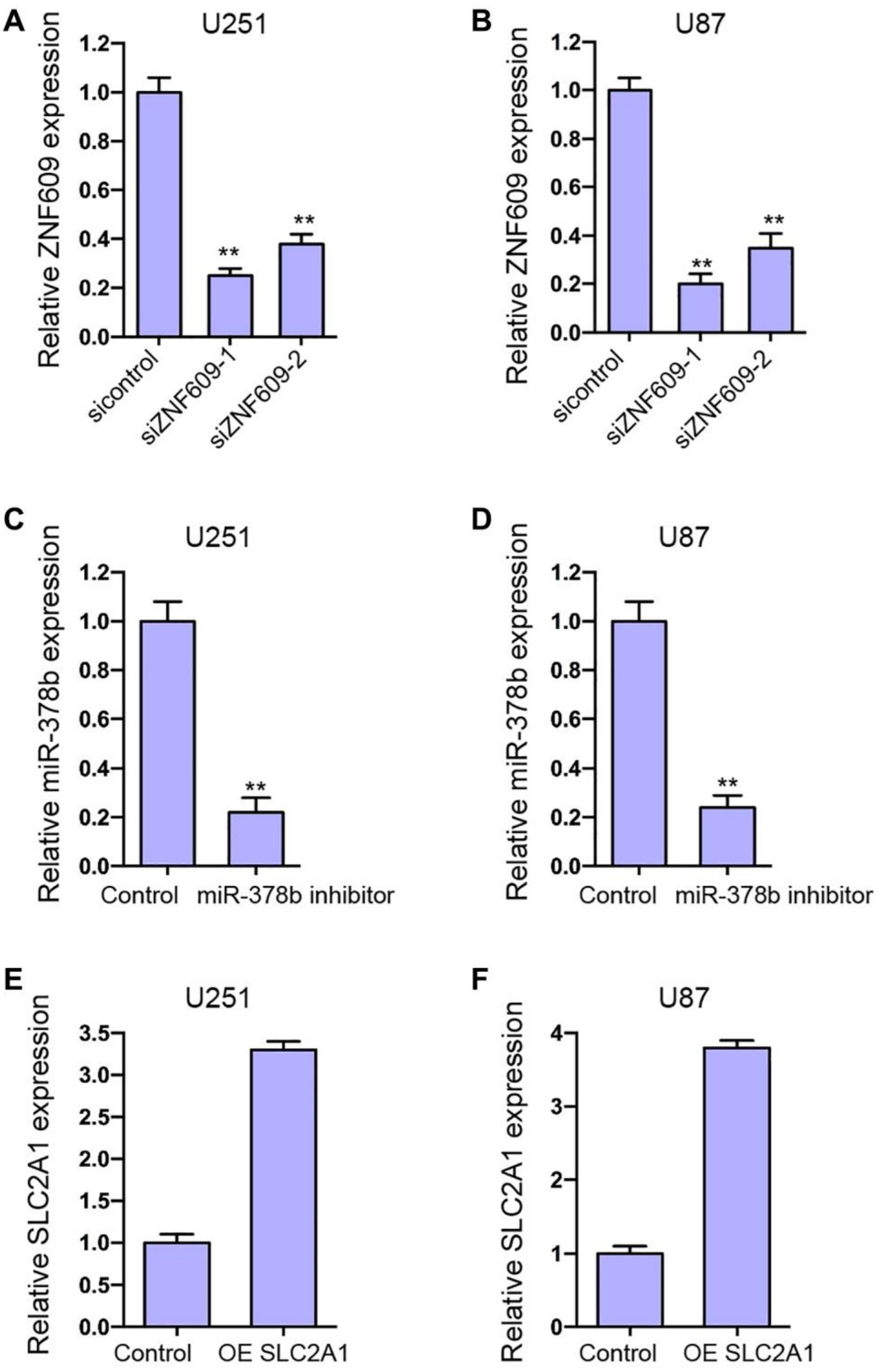


**Supplementary Figure 1. The validation of ZNF609 depletion, miR-378b inhibition, and SLC2A1 overexpression.** (**A** and **B**) The effectiveness of ZNF609 siRNAs was verified by qPCR in U251 and U87 cells. (**C** and **D**) The effectiveness of miR-378b inhibitor was verified by qPCR in U251 and U87 cells. (**E** and **F**) The effectiveness of SLC2A1 overexpression was verified by qPCR in U251 and U87 cells. mean ± SD, $^{**}P < 0.01$.
